# Supplementary material for: Migratory Tumor Cells Cooperate with Cancer Associated Fibroblasts in Hormone Receptor-Positive and HER2-Negative Breast Cancer
Source: Int J Mol Sci. 2024 May 28;25(11):5876. doi: 10.3390/ijms25115876 (PMC11172245; doi:10.3390/ijms25115876)
Supplement: Supplementary file 1 [file ijms-25-05876-s001.zip › ijms-2948981-supplementary(3)/supp_figures_1_10_rev1.pdf]

Fig. S1

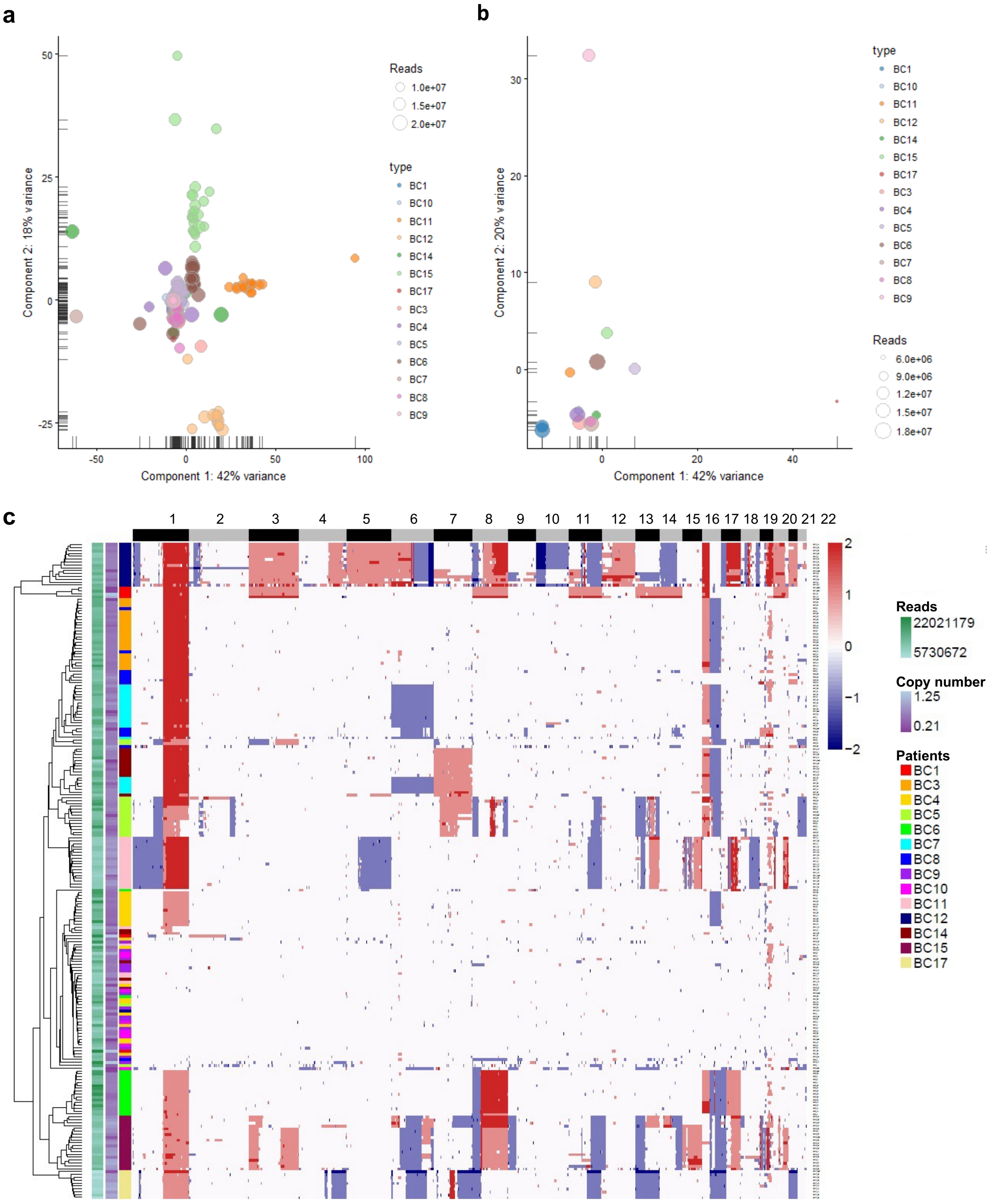

Fig. S1 Quality assessment of SIDR-WGS compared to bulk WGS (a) PCA plot showing the read quality of the 228 SIDR-WGS data from 14 patients. **(b)** PCA plot showing the read quality of 14 bulk WGS data. **(c)** Heatmap showing the copy number variation of 14 samples.

Fig. S2

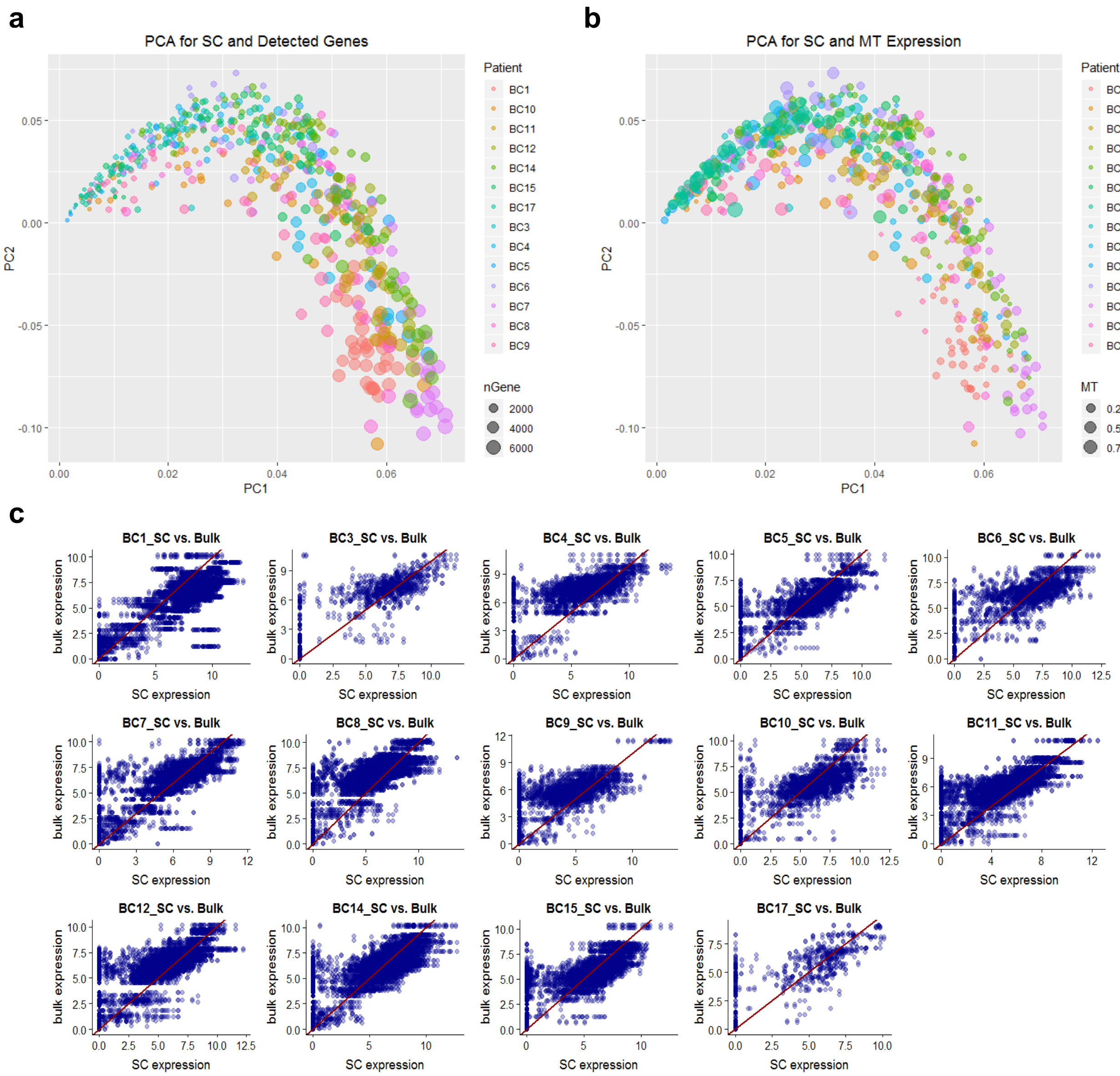

Fig. S2 Quality assessment of SIDR-WTS compared to bulk WTS (a) PCA plot showing number of detected gene of 408 SIDR-WTS from 14 patients. **(b)** plot showing percentage of mitochondrial gene in each cell **(c)** Gene expression level comparison between SIDR-WTS and bulk RNA-seq in 14 individuals.

Fig. S3

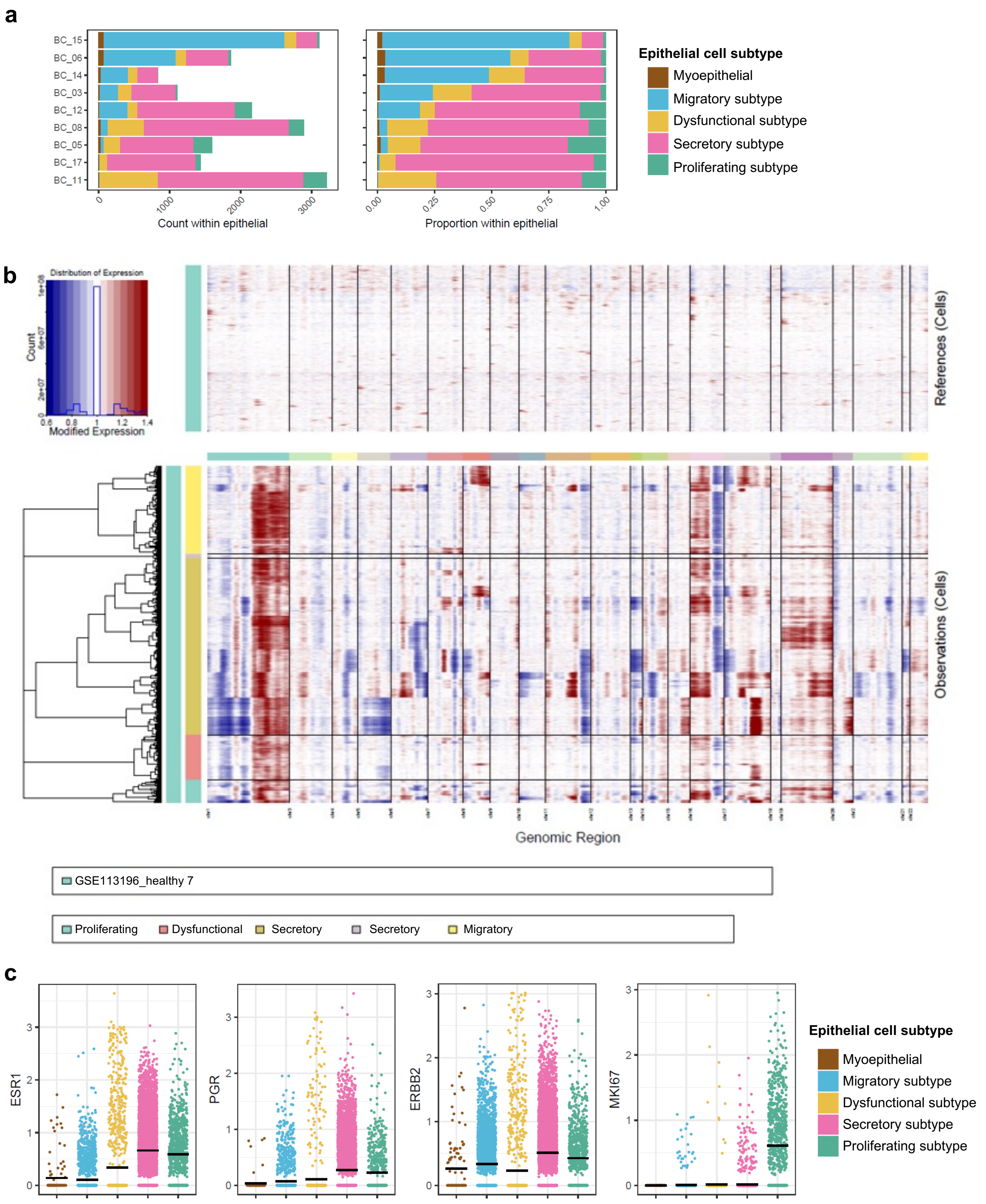

Fig. S3 Characteristics of tumor epithelial cell of HR+/HER2- breast cancer (a) Proportion of tumor epithelial cells and myoepithelial cells across individuals. (b) Inferred copy number changes of epithelial cell compared healthy control sample no. 7 from GSE 113196(1) using “inferCNV”. (c) Breast cancer biomarker expression in epithelial cell subtypes compared to myoepithelial cell.

Fig. S4

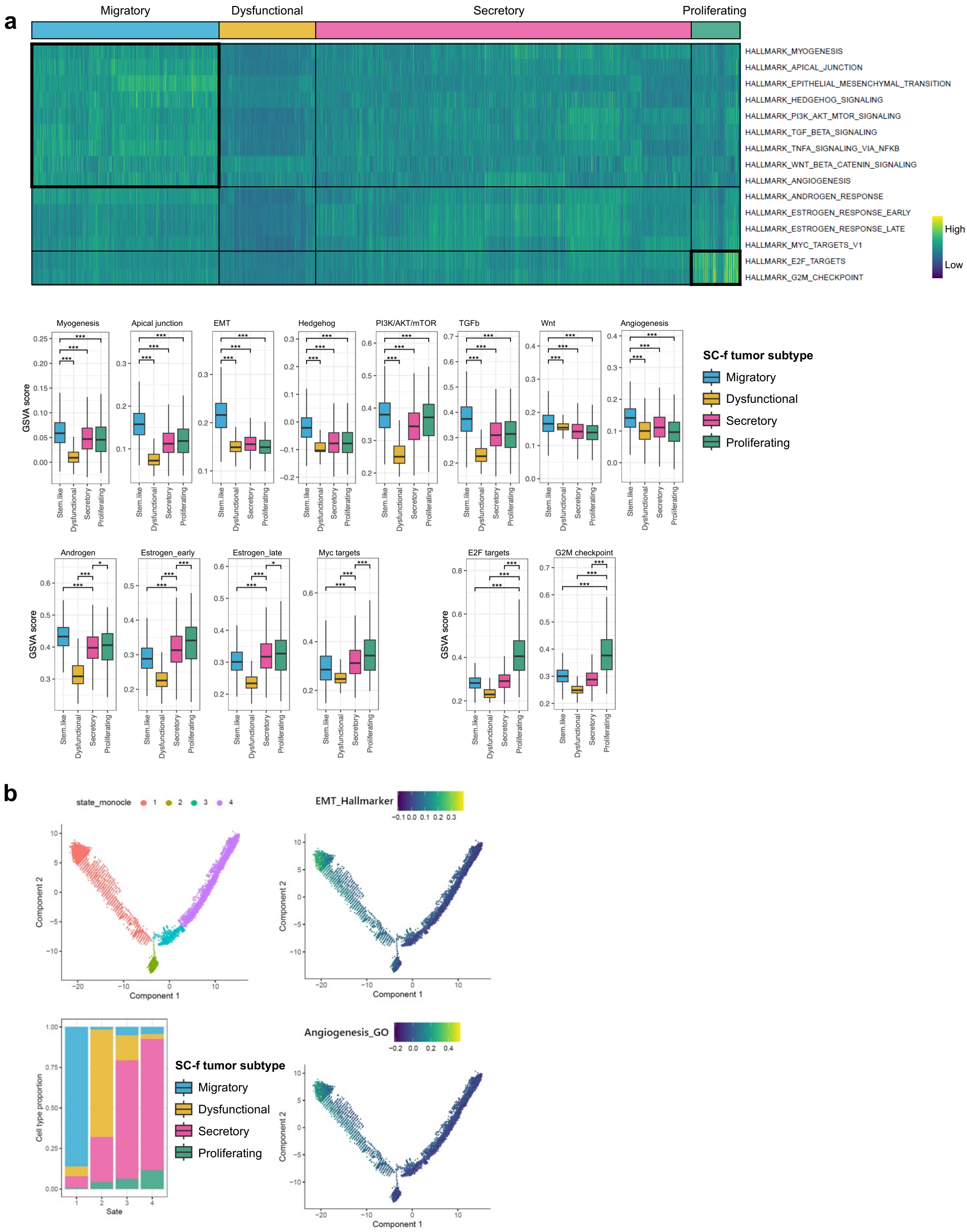

Fig. S4 Trajectory analysis of HR+/HER2- breast cancer (a) Heatmap and boxplots of single-cell GSVA score using HALLMARK gene sets. (b) Trajectory analysis showing 4 states according to branch status and their composition of SC-f tumor subtype (left). EMT and angiogenesis features were observed in early pseudotime (right).

Fig. S5

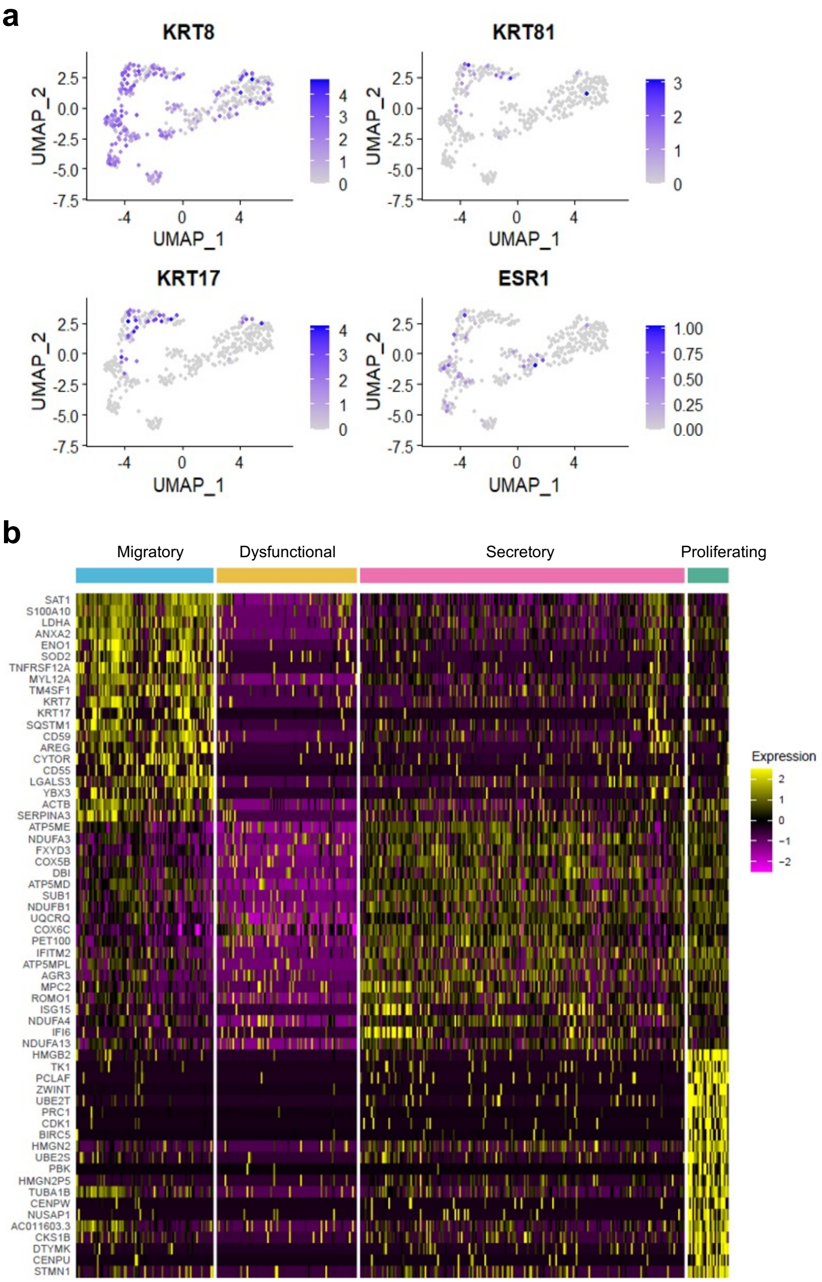

Fig. S5 Tumor epithelial classification in SIDR-WTS (a) UMAP plot of 408 SIDR-WTS cells indicating epithelial markers (KRT8, KRT81 and KRT17) and ESR1 expression (b) DEGs of each SC-f tumor subtype in SIDR-WTS.

Fig. S6

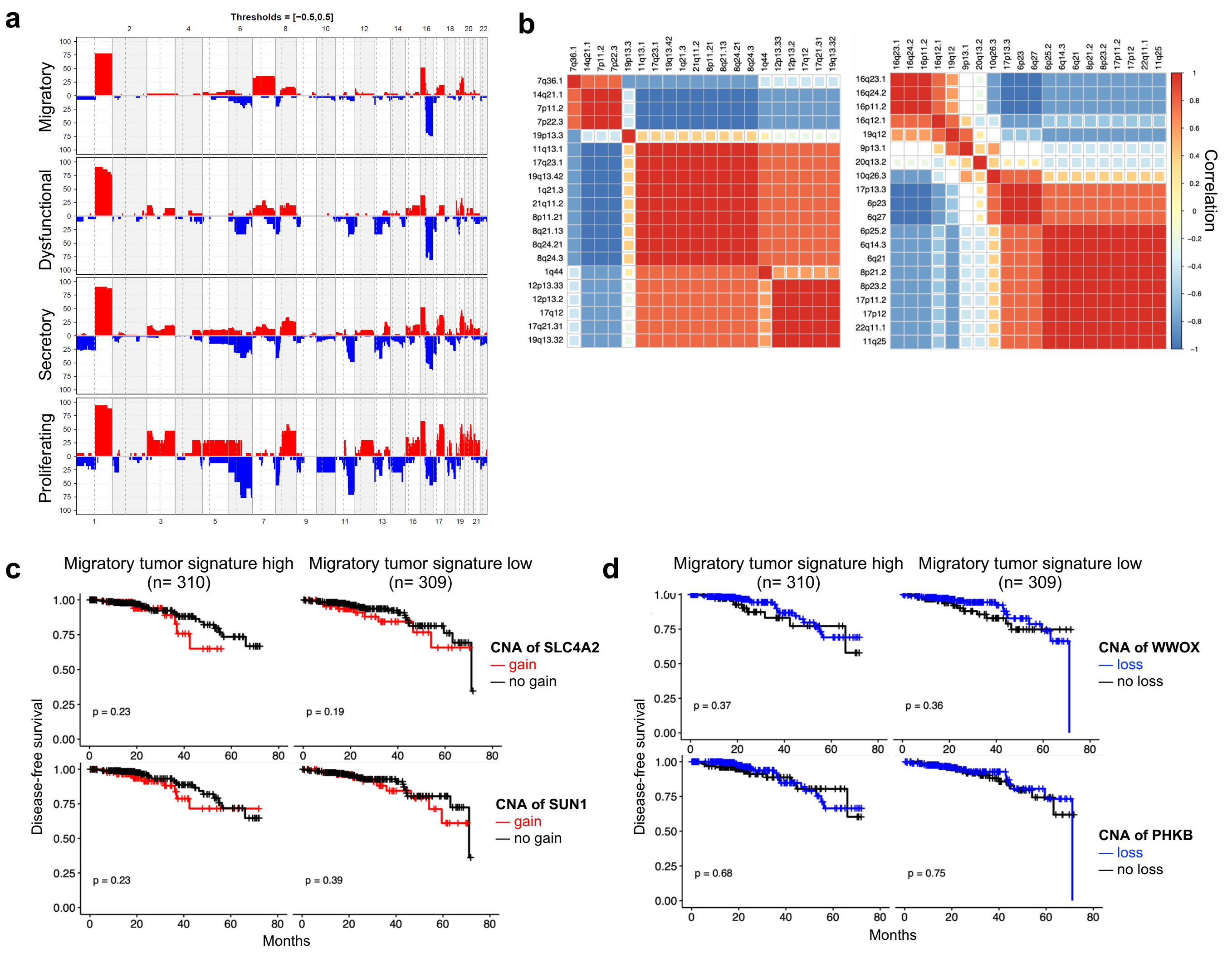

Fig. S6 CNV aberrations in SIDR-WGS (a) Total frequency of copy number alteration occurred in each SC-f tumor subtypes. (b) Correlation of the focal CNA aberration pattern in SIDR-WGS of gains (left) and losses (right). (c),(d) Two-factor Kaplan-Meier survival analysis for 619 hormone receptor positive TCGA-BRCA patients according to the CNA gain/loss and calculated migratory subtype feature signature score.

Fig. S7

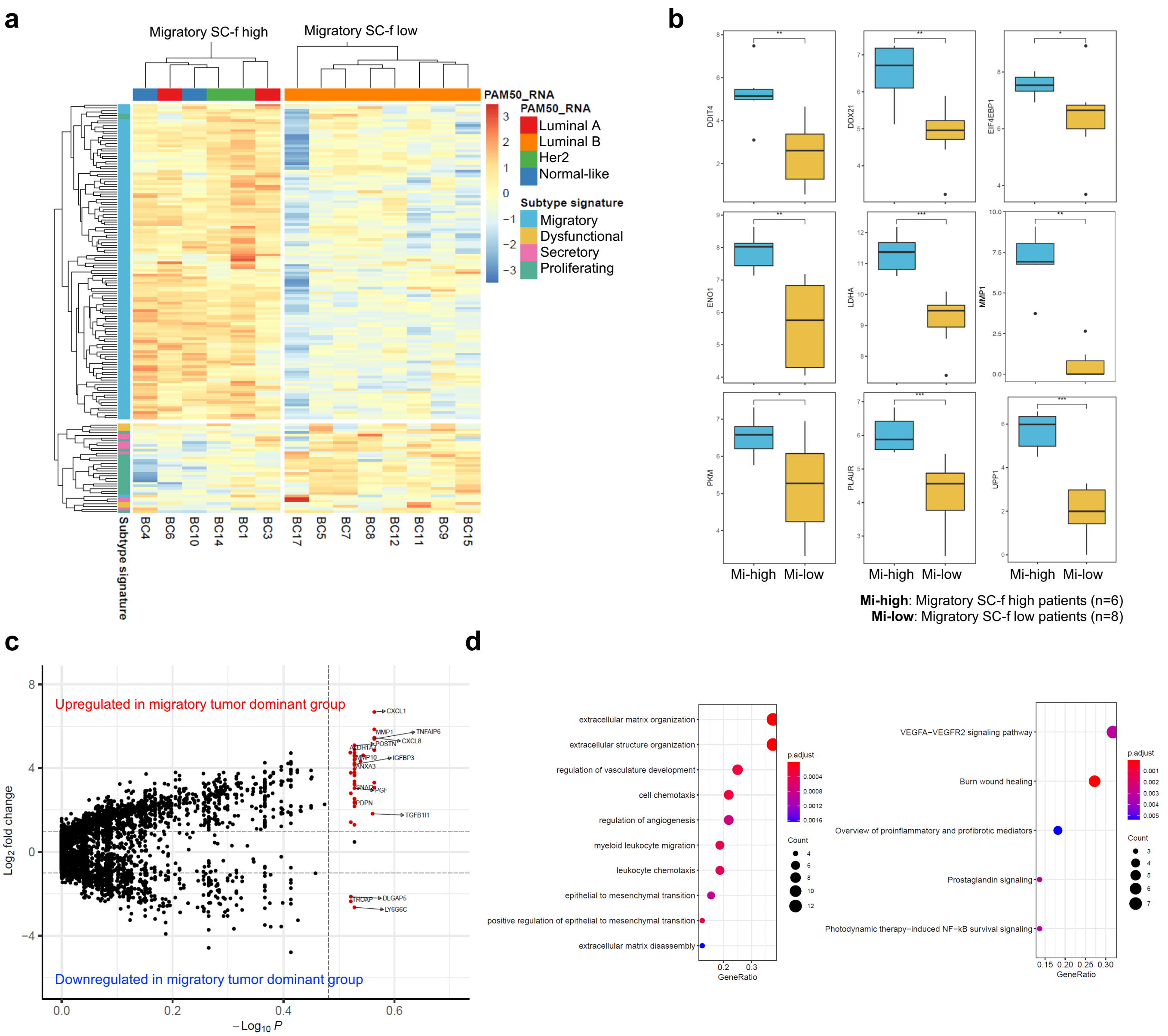

Fig. S7 SC-f tumor subtype reproduced in bulk RNA-seq (a) Classification of 14 HR+/HER2-BC patients into SC-f tumor subtypes. They divided only two groups, migratory and others. (b) Comparison of migratory tumor signature gene expression from 10x scRNA-seq analysis (c) DEGs between migratory and other group. (d) Pathways enriched in migratory tumor group, left based on GO and right based on KEGG pathway.

Fig. S8

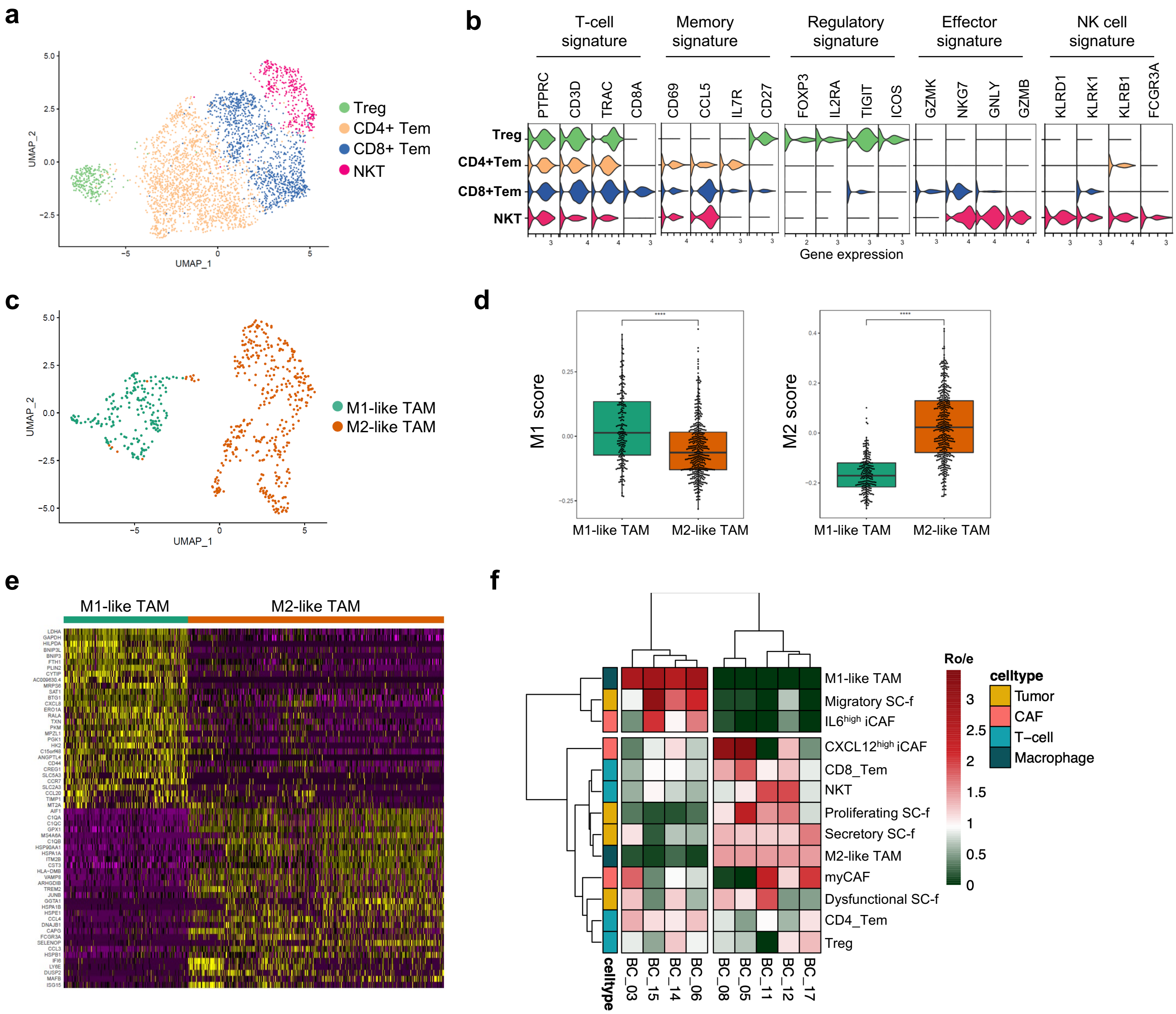

Fig. S8 Tumor microenvironmental cell composition of HR+/HER2- breast cancer (a) UMAP plot of 3,515 T-cells of HR+/HER2-BC representing four subpopulations from scRNA-seq. (b) Expression of T-cell subtype markers. (c) UMAP plot of 593 macrophage cells of HR+/HER2-BC representing two subpopulations. (d) M1 TAM and M2 TAM score(2) comparison of HR+/HER2-BC TMAs (e) DEGs between M1-like and M2-like TAM. (f) Ratio of observed to expected cell numbers (Ro/e) of tumor and microenvironmental cells by each patients.

Fig. S9

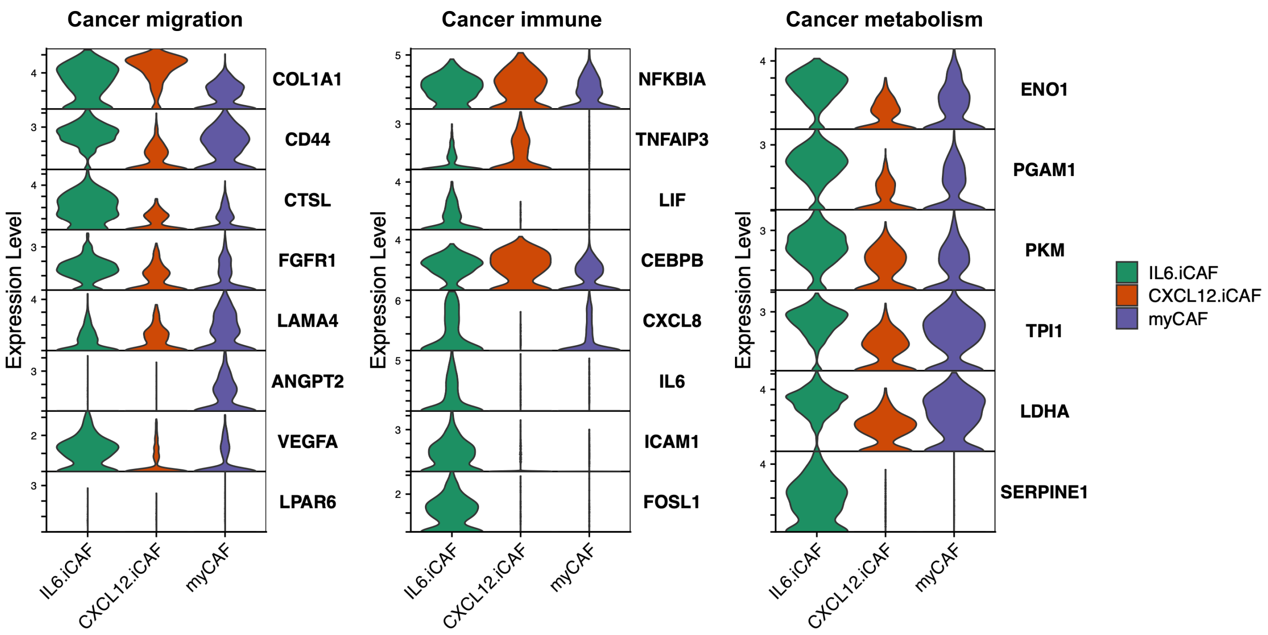

Fig. S9 Functional gene expression in CAFs of HR+/HER2- breast cancer Violin plot showing expression of functional genes in HR+/HER2-BC CAFs.

Fig. S10

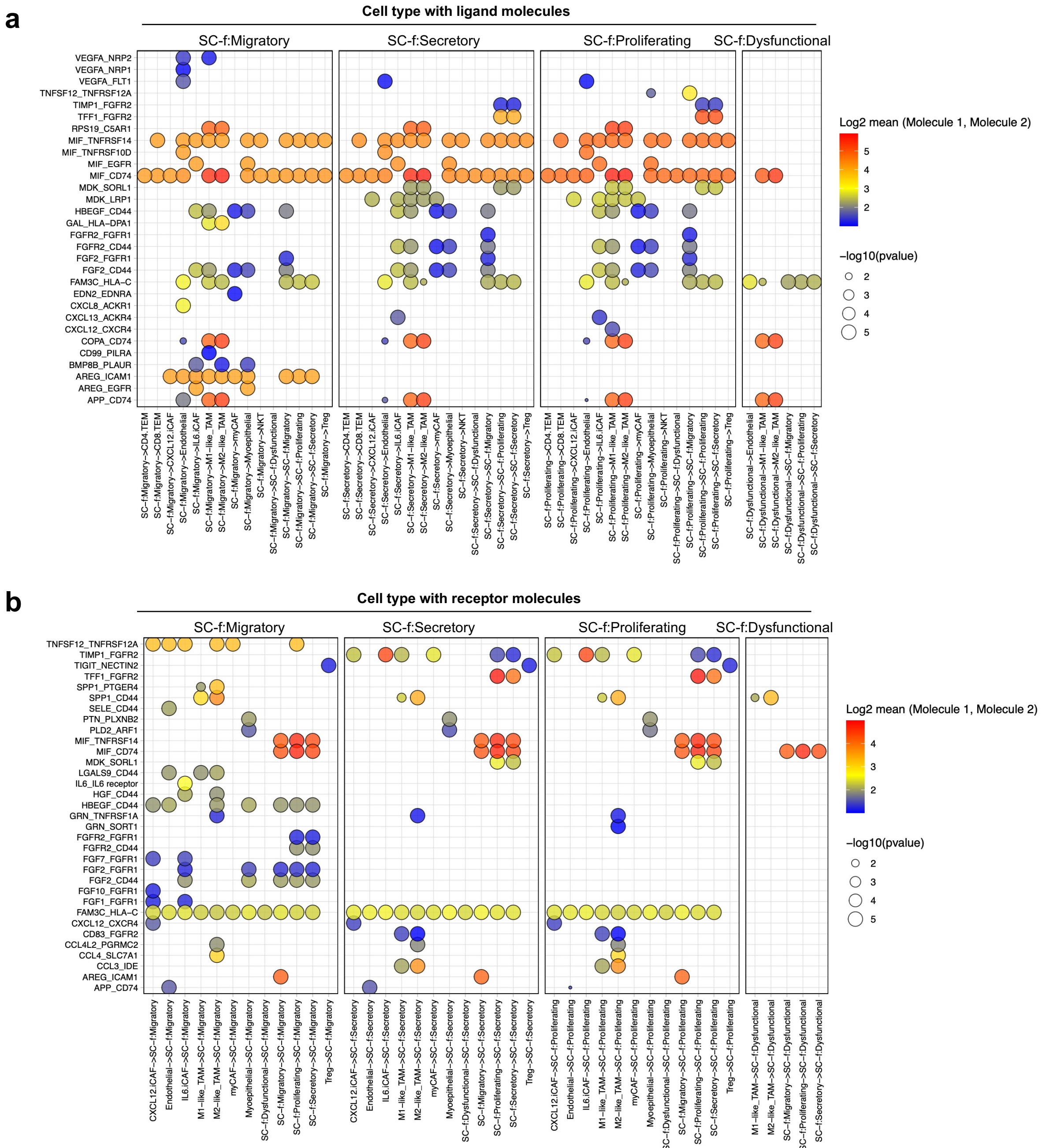

Fig. S10 Ligand and receptor interaction between tumor cells and TME cell types (a) Dot-plot showing the prevalence and expression of paired ligand and receptor interaction from four types of SC-f tumor subtype toward each TME cell type. (b) Dot-plot showing the prevalence and expression of paired ligand and receptor interaction from TME cell types toward each four types of SC-f tumor subtype.
